# Black Nurses Collaborative Approach to Addressing COVID-19 in Black Communities

**DOI:** 10.1007/s40615-021-00987-9

**Published:** 2021-02-12

**Authors:** Martha A. Dawson

**Affiliations:** 1 The National Black Nurses Association, Inc., Silver Spring, MD USA; 2 Department of Family, Community and Health Systems, The University of Alabama at Birmingham School of Nursing, Birmingham, AL USA

**Keywords:** COVID-19, Social determinants, Black nurses, Inequity, Health model

## Abstract

As with other national disasters, epidemics, and pandemics, the novel coronavirus SARS-CoV-2 (COVID-19) pandemic has highlighted health disparities in Black communities in the USA. Healthcare providers, community activists, politicians, members of faith-based organizations, professional athletes, and Black families are asking crucial questions about why Black and Brown people are disproportionately infected by, and dying from, the COVID-19. Evidence in healthcare and social sciences literature demonstrates that historically, systemic racism and injustices play a large role in the health and well-being of Blacks living in the USA. For decades, the National Black Nurses Association has been on the forefront, engaging our people using a collaborative community-based practice model. The healthcare goal in the USA should center on health protection, promotion, and prevention, moving toward a wellness model and away from treatment of illnesses that contribute to healthcare waste. Finally, awareness of social determinants of health has taken center stage, demonstrating how laws, policies, and practice affect health outcomes and the well-being of Black and Brown communities. In order to address social determinants of health and healthcare inequity, the National Black Nurses Association has called for an increase in the number of Black registered nurses and licensed vocational and practical nurses. The healthcare goal in the USA should center on health protection, promotion, and preventions moving toward a wellness model and away from treatment of illnesses that contributes to healthcare waste.

## National Black Nurses Association Introduction

The National Black Nurses Association (NBNA) was organized in 1971 under the leadership of Dr. Lauranne Sams, former dean and professor of nursing, school of nursing, at Tuskegee University in Tuskegee, Alabama. NBNA, was incorporated in 1972 in the state of Ohio, is the professional voice for African American-registered nurses, licensed vocational/practical nurses, nursing students, and retired nurses [1]. The organization includes 114 chapters in 33 states and the District of Columbia, Washington, DC. NBNA is a non-profit professional organization that promotes community service, health policy and advocacy, workforce expansion, and professional development.

For 49 years, NBNA nurses and nursing students have worked to make a difference in the quality of life in our communities by providing culturally competent and congruent health services where Black and Brown people live, work, worship, and play. NBNA uses a community partnership approach to engage the Black population in addressing healthcare needs and outcomes. The organization also collaborates with other like-minded private and public agencies to effect healthcare changes through community service and education. During the novel coronavirus SARS-CoV-2 (COVID-19), Black nurses, while risking their personal safety and the health and well-being of their families, are serving as essential frontline workers and are providing direct care to patients in hospitals, nursing homes, and long-term care facilities, risking their and their families lives. These nurses also served as the most trusted source of information to help the Black community address the many myths and lack of information accuracy about COVID-19. Community-based services and education are the hallmark of NBNA’s role in improving access to care and improved health outcomes in the Black population. NBNA members were working in their communities prior to COVID-19 providing preventative health screenings and health education on high blood pressure, blood glucose, cholesterol, HIV, cancer, sickle cell disease, and mental health.

## Collaborative Community Health Model

In the 1991, NBNA leaders received funding from the US Department of Health and Human Services Division of Nursing to develop a collaborative community health model (CCHM) [2]. The CCHM provides a framework to deliver structured and measurable programs to address health disparities and to improve the health status of the Blacks and other minority populations. Using the blueprint of Healthy People (HP) 2000 and subsequent HP2010, HP2020, and HP2030, the model provides a basis for addressing health protection and promotion, disease prevention, surveillance, education, and data management. In addition, the CCHM has an embedded community-based active engagement approach that support collaborative partnerships with advocacy groups, corporations, health systems, schools, universities, and governmental agencies. This model is also grounded in the concept of community inclusion, social justice, human caring, and healthcare as a fundamental human right.

## COVID-19 and Social Determinants of Health (SDOH)

COVID-19 continues to spread in the USA with seven million positive cases on 9/27/2020 and a reported 311,102 cases over a 7-day period. As of the end of September 2020, the total number of deaths associated with COVID-19 in the USA is 204,033 [3]. With the COVID-19 pandemic, a disproportionate number of African American/Blacks (AA) are contracting and dying from the virus. AAs account for about 13.4% of the US population [4] and 18.3% of COVID-19 cases and 20.9% of the deaths [5]. The data seem to support a higher prevalence of COVD-19 in AAs with subsequent poor survival rates. Early during the COVID-19 pandemic, Black and Brown social activists; clinical, translational, and social scientists; politicians; and others began discussing the roles of health disparities and how social determinants of health were impacting outcomes in populations of color. The CDC [6] defines SDOH as societal and environmental conditions where people are born, grow up, live, work, play, worship, age, and transition life. SDOH include such factors as housing, food insecurity, transportation, education, social support, employment, income, social status, and racism and discrimination [4, 6].

During each national disaster such as hurricanes, tornadoes, or dangerous outbreaks of diseases, the USA returns to the same health disparities conversation and debate. Here lies the problem; the country engages in conversation but fails to develop and implement an action plan. After Hurricane Katrina, the health disparity conversation ended almost as fast as the water receded from the Ninth Ward in New Orleans. However, the same SDOH remained post the disaster. Post-COVID-19, the conversations about the living conditions of “essential employees” must continue. The USA needs an action plan to improve the lifestyle, environment, education, income, and access to healthcare of every citizen.

Activists, politicians, and every day citizens must demand that the US health system moves from an illness and treatment model to a health protection and prevention model. The USA wastes billions of dollars every year. In 2019, researchers estimated that the waste accounts for 30% of total healthcare spending, and the waste is divided into six categories: (1) failure of care delivery, (2) failure of care coordination, (3) overtreatment, (4) pricing failure and low-value care, (5) fraud and abuse, and (6) administrative complexity [7]. When one considers the illness model of care that the US uses (focus on treatment and cures) and the estimated $700 to $900 billion in waste each year, it becomes apparent as to why our responses and ability to aggressively contain COVID-19 are falling short. When it comes to the Black and Brown communities, zip codes matter [4, 5, 8]. COVID-19 is a respiratory illness with an incubation period meaning that a person can be infected with the virus prior to experiencing and exhibiting symptoms [9]. COVID-19 spreads via contact and droplet from coughing. Therefore, one of the recommended treatments is isolation from others. When a person is living in poverty in poor housing and multi-generational families, the preventive measure of isolation is impossible. Black and Brown “essential workers” must use mass transportation, and early during this pandemic, they were not provided personal protective equipment which put workers and their families at risk. This pandemic has clearly exposed the fact that race, ethnicity, age, gender, zip code, education, income, and power and politics matter in the US healthcare system. COVID-19 data show that at-risk-population include those with pre-existing conditions, age 65 or older, males, healthcare workers, and other essential workers [9].

## COVID-19 and NBNA Response

Since NBNA has an ongoing presence in the community and a large network of partners, we were one of the first to start informing and educating our communities by working with faith-based organizations, politicians, social service agencies, healthcare systems, public health departments, and many others. NBNA’s Collaborative Community Health Model is used as the practice framework by nurses in our 114 chapters to reach out to other national organizations such as the National Urban League, National Medical Association, National Dental Association, Association of Black Cardiologists, Rainbow Push Coalition Health Committee, One Hundred Black Men of America, National Bar Association, and the National Council of Negro Women. NBNA has collaborated with Black Women for Positive Change, Centers for Disease Control and Prevention, Black Congressional Caucus, and the Black Women’s Health Imperative to reach Black and Brown citizens to ensure they are informed and educated about COVID-19. These relationships are important to advocacy role that NBNA plays in shaping health policy.

NBNA provides educational webinars, workshops, and conferences. NBNA media footprint has expanded to include op-ed, news releases, and appearances on television and radio. The national office staff and local chapter members are working in advisory roles and on committees with companies that are in the healthcare space such Pfizer, VITAS Healthcare, CVS Health, Prolacta, Novartis, Abbott, Johnson & Johnson, UnitedHealth Group, and Gilead and hospitals such as Children’s Mercy Kansas City. Through these networks, NBNA is able to sit at the decision-making table regarding research, clinical trials, and resource distribution.

With regard to COVID-19, new partners have joined us in meeting the needs of front-line workers. When NBNA members needed personal protective equipment for our members and resources in our communities, partners such as PUMA, DTLR, Clove, Diddy Love Team, Careismatic Brands, Soul for Soles, Daisy Foundation, and Direct Relief joined NBNA’s team. Through the efforts of these companies, NBNA provided face masks, shoes, shoe covers, gloves, gowns, caps, thermometers, and pulse oximeters to frontline workers. COVID-19 care kits were provided to patients and community members. In addition, NBNA members participated in food drives, provided meals for essential works, created safety rounds for checking on home-bound elders, and found time to support the *Black Lives Matter* movement and to care for protesters. Then, we had partners such as the Black Hollywood Education and Resource Center and other entertainers that provided mental help breaks for NBNA’s members to relax, refresh, and provide self-care. NBNA partner with Pfizer and FirstResponderFirst to provide free counseling and mental health services to nurses across the country, including non-NBNA members. The organization through its 114 chapters is planning additional workshops and activities as COVID-19 and the flu season begin creating a twin pandemic.

Healthcare in the USA is a big business and one of fastest growing job industries. It is estimated that seven in every10 jobs in the USA are related to healthcare. However, there is an under representation of AA in healthcare professional positions such as medicine, dentistry, nursing, pharmacy, and other health specialties. There are 3.9 million registered nurses (RN) in the USA. AA is only 7.8% of the US RN population [10]. Nursing is a science-based discipline, and potential students need a strong foundation in science, technology, and math. Due to poorly funded education systems in many AA communities, many students are unprepared for the academic rigor of healthcare-related curricula. NBNA is addressing this issue by working with elementary, junior high, high school, and pre-nursing college students. The organization’s goal is to increase the number and percentage of AA registered nurses to 11% or higher of the total RN population. Workforce expansion is a key strategic focus of NBNA. NBNA’s founding members recognized that in order to make a difference in the quality of life in their communities that black nurses across the nation had to take the lead [1]. Therefore, we are on the frontline fighting structural and institutional racism and injustices.

## Conclusion

The COVID-19 pandemic has moved the USA closer to the edge of chaos. In the midst of this healthcare crisis, there are societal issues around systemic and institutional racism that expose how law and policy in the USA contribute to the SDOH and poor health outcomes. It is in our hands to seize this moment and become innovators of solutions or remain bystanders of injustice. The USA as a nation cannot continue to quarantine and relegate our conscious with silent voices. The USA should have learned from World Wars I and II, the Vietnam War, Jim Crow laws, the HIV/AIDS epidemic, and other man-made and natural disasters that justice, equity, and human caring matters. The call to action is: What will the US do differently this time? What lessons will the political powers and scientists embrace, and what visible, measurable changes will the people demand? Most importantly is how will business, hospitals, healthcare industry, and educational institutions lead this change. Change must come because COVID-19 has shown us that we are all vulnerable, either directly or indirectly.

## Data Availability

Not applicable
